# Intra-Articular Application of Sluijter-Teixera Poisson Pulsed Radiofrequency in Symptomatic Patients with Knee Osteoarthritis: Focus upon Clinical Efficacy and Safety

**DOI:** 10.1155/2021/5554631

**Published:** 2021-02-20

**Authors:** D. Filippiadis, A. Tsochatzis, E. Petsatodis, S. Galanis, G. Velonakis, C. Giankoulof, A. Kelekis

**Affiliations:** ^1^2nd Department of Radiology, University General Hospital “ATTIKON,” Medical School, National and Kapodistrian University of Athens, Athens, Greece; ^2^Interventional Radiology Department, George Papanikolaou General Hospital, Thessaloniki 57010, Greece

## Abstract

**Purpose:**

To retrospectively evaluate the effectiveness of intra-articular application of Sluijter-Teixera Poisson pulsed radiofrequency (STP PRF) in knee osteoarthritis symptomatic patients with chronic pain refractory to conservative therapies.

**Materials and Methods:**

Institutional database research of two centers identified 39 cases of knee osteoarthritis patients treated with intra-articular STP PRF. Pain prior and one-week and one-, three-, six-, and twelve-month post-STP PRF was compared by means of a numeric visual scale (NVS) questionnaire. Cardiovascular and Interventional Radiological Society of Europe (CIRSE) classification system was used for complications reporting. Mean patient age was 71.59 ± 11.99 years, mean body mass index was 30.23 ± 4.69, and male/female ratio was 9/30.

**Results:**

Mean baseline pain score was 8.31 ± 1.70 NVS units. This was reduced to a mean value of 0.90 ± 1.50 NVS units one-week post-RF, 1.08 ± 1.53 at one month, 1.54 ± 1.88 at three months, 2.33 ± 2.17 at six months, and 3.23 ± 2.23 at 12 months of follow-up (*p* < 0.01). Pain decrease of more than 4 NVS units was noticed in 35/39 knees (89.7%) at first week, 36/39 knees (92.3%) at first month, 35/39 knees (89.7%) at three months, 32/39 knees (82.1%) at six months, and 25/39 knees (64.1%) at one year. There was no recurrence during the follow-up. No complication was observed.

**Conclusions:**

Percutaneous, intra-articular application of STP PRF is an effective and safe technique for chronic pain reduction in patients with knee osteoarthritis. Results seem to be reproducible and long lasting with significant patient satisfaction at 12-month follow-up.

## 1. Introduction

The most common cause of chronic knee pain is degenerative osteoarthritis most commonly affecting middle-aged and elderly patients resulting in significant functional capacity reduction [1, 2]. Therapeutic armamentarium includes physical and oral pharmacologic therapy, intra-articular injections, neurolytic or neuromodulatory techniques, transcatheter arterial embolization, minimally invasive arthroscopic treatment, and partial or total knee arthroplasty [3–6]. Intra-articular application of pulsed radiofrequency with or without viscosupplementation has been reported in different studies as a safe and efficacious technique for pain reduction and mobility improvement in symptomatic patients suffering from degenerative knee osteoarthritis [7–9]. Pulsed mode of radiofrequency energy (PRF) deposition is characterized by long silent phases (480 milliseconds) which between the short bursts of energy application (10–20 milliseconds) contribute to maintaining tissue temperature under 42°C which is below the irreversible tissue damage threshold; this results in much less (if any) neurodestructive potential [10, 11]. PRF creates a neuromodulatory effect, suppressing both excitatory C-fibers activation and the spread of pain impulse at the synaptic junction, in addition to a modulatory effect on proinflammatory cytokines [6, 9].

A variety of pulsed radiofrequency mode utilizes the Poisson curve for energy distribution (Sluijter-Teixera Poisson radiofrequency) (STP) aiming to provide pulses which are meticulously spread in order to achieve highest treatment result with the lowest heat development [12–14]. STP mode of pulsed radiofrequency provides a short pulse width for minimal destructive effect and a higher coefficient of variance for better treatment effectiveness. This variation in pulsed mode has been applied inside the intervertebral discs for discogenic pain and intra-articularly for arthrogenic pain with preliminary results reporting significant efficacy rates on terms of pain reduction and mobility improvement [12–14]. Combining intra-articular application of pulsed radiofrequency to genicular nerve pulsed neuromodulation seems to result in improved WOMAC (Western Ontario and McMaster Universities Arthritis Index) scores at 3 months after the treatment with a longer period of efficacy when compared to extra-articular application alone; however, in this first clinical comparative study of different approaches for pulsed radiofrequency in knee osteoarthritis, both arms were effective in reducing pain at 3 and 6 months follow-up [15]. Intra-articular application of RF in the knee joint is related to the action of electric fields on immune cells rather than on deflection of the current by bony surfaces and therefore could work as a stand-alone approach.

The purpose of this study is to retrospectively evaluate the effectiveness of intra-articular application of Sluijter-Teixera Poisson pulsed radiofrequency in patients with knee osteoarthritis suffering from chronic pain refractory to conservative therapies.

## 2. Materials and Methods

### 2.1. Patient Selection and Evaluation

Institutional database research of two centers from 01/12/2018 to 01/08/2020 identified 39 symptomatic patients with knee osteoarthritis who underwent intra-articular application of STP PRF. Inclusion criteria included adult patients with symptomatic knee osteoarthritis diagnosed with X-rays and classified as grade II to IV according to the Kellgren–Lawrence (KL) classification; pain in all patients was located at the level of the knee joint with no neurologic signs and was refractory to conservative therapies (analgesics and nonsteroidal anti-inflammatory drugs as well as physiotherapy) in the past six months without success. At the time of treatment, all patients had discontinued all drug therapy for at least two weeks. All included patients and lesions should have been evaluable for the 12-month follow-up. The diagnosis was made by an interventional radiologist with 11 years of experience or the referring orthopaedic surgeon who identified the potential participants and verified their eligibility. Preoperational evaluation included imaging with knee X-rays on anterior-posterior and lateral views used to evaluate patients according the Kellgren–Lawrence (KL) classification along with clinical evaluation; from the 39 patients included in the present study, 7 were classified as grade 2 (KL-2), 18 as grade 3 (KL-3), and 14 as grade 4 (KL-4). Exclusion criteria for the procedure included untreatable coagulopathy, active, systemic, or local infections and patient unwilling to consent to the procedure and the study.

### 2.2. Technique

**Figure 1:**
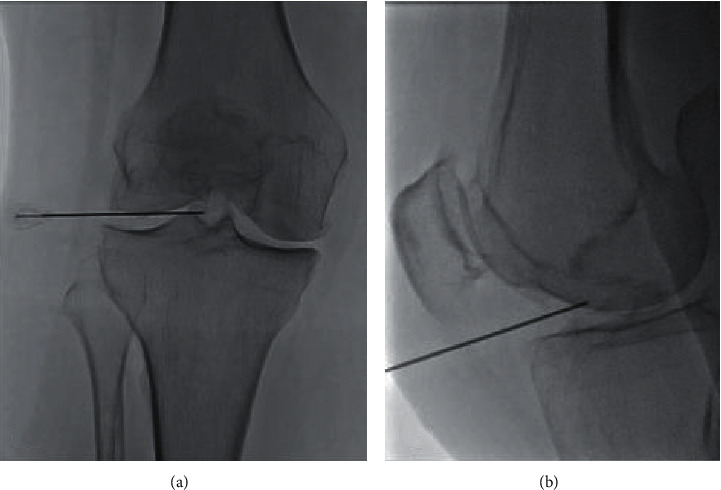
(a) Face fluoroscopy view illustrating the final position of the trocar at the level of the tibial crest. (b) Lateral fluoroscopy view illustrating the final position of the trocar anteriorly to the tibial crest.

Under extensive local sterility and fluoroscopic guidance, selection of the entrance skin point was performed and local anesthesia (3–5 ml of lidocaine hydrochloric 2%) was applied. No preoperative antibiosis was intravenously administered. A 20 gauge/10 cm RF cannula was percutaneously inserted from the anterolateral region of the knee joint. The final position of the RF cannula inside the joint (midline and in an equidistant level between tibial and femoral bones) was fluoroscopically verified in face and lateral projections (Figure 1). Coaxially, a RF electrode with a 10 mm “active tip” (EQUIP MEDIKEY BV, Gouda Netherlands) was introduced, and a 10-minute neurolysis session was performed with PRF (1,200 pulses at 50 V with 10 ms duration followed by a 480 ms silent phase). Each patient remained in the hospital for 30–45 minutes (only for observation) and was then discharged with suggestions of 1-day rest and then being free to engage in normal activities.

### 2.3. Statistical Analysis

Continuous variables are presented as mean ± SD, whereas categorical variables are presented as absolute frequencies. Pain prior and one-week and 1-, 3-, 6-, and 12-month post-STP PRF was compared by means of a numeric visual scale (NVS) questionnaire [16]. To evaluate differences from baseline to post-RF follow-up, a 3 × 5 (osteoarthritis group by time) mixed model analysis of variance (mixed model ANOVA) was conducted (with osteoarthritis group as the between-group factor and time as the within-group factor). Significant main effects for time were followed by dependent *t*-tests between baseline and follow-up timepoints. Statistically significant group by time interactions was further explored using pairwise comparisons. Pain improvement and recurrences were defined according to previous study [9]. Specifically, improvement was defined as any pain decrease of more than 4 NVS units after the treatment. Recurrence during the follow-up was defined as any pain increase lower than the score before treatment despite initial improvement. The association between the percentage of cases with improvement/no change/recurrence and osteoarthritis groups was examined using chi-square (*χ*^2^). The statistical threshold was set at *p* < 0.05, with Bonferroni correction for multiple comparisons. All analyses were conducted with SPSS v. 22.0 (IBM Corp, Armonk, NY). The definition of complications was assigned according to the Cardiovascular and Interventional Radiological Society of Europe (CIRSE) classification system [17].

## 3. Results

**Figure 2:**
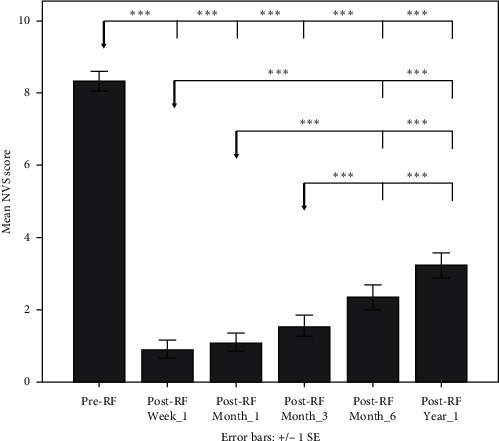
Bar chart illustrating mean NVS scores and standard error (1SE) prior and during the follow-up period across all groups (note: the reference point for each comparison is indicated with a grey arrow; ^*∗∗∗*^*p* < 0.005, ^*∗∗*^*p* < 0.01, and ^*∗*^*p* < 0.05 after Bonferroni correction).

Table 1 presents demographic and patient-related characteristics for the total sample of 39 cases. Descriptive measures (mean, SD, and Min-Max) for NVS were calculated both at baseline before RF and different follow-up timepoints. The profile of mean NVS score at baseline and follow-up timepoints is shown in Figure 2. Mean pain score prior to RF was 8.31 ± 1.70 NVS units. This baseline score was reduced to a mean value of 0.90 ± 1.50 NVS units one-week post-RF, 1.08 ± 1.53 NVS units at one month, 1.54 ± 1.88 NVS units at three months, 2.33 ± 2.17 NVS units at six months, and 3.23 ± 2.23 NVS units at 12 months of follow-up (Table 2).

A 3 × 5 mixed model ANOVA with Greenhouse–Geisser correction showed a significant main effect of time [*F* (3.017, 108.613) = 179.577; *p* < 0.001, partial eta^2^ = 0.833)], osteoarthritis group [*F* (2, 36) = 12.947; *p* < 0.001; partial eta^2^ = 0.418], and osteoarthritis group by time interaction [*F* (6.034, 108,613) = 4.116; *p*=0.001; partial eta^2^ = 0.186]. Paired samples *t*-tests with Bonferroni correction were used to examine post hoc comparisons on NVS between different follow-up timepoints across all osteoarthritis groups (Figure 2). We found significant differences between baseline and (a) week_1 NVS (mean difference = 7.271; *p* < 0.001), (b) month_1 NVS (mean difference = 7.051; *p* < 0.001), (c) month_3 NVS (mean difference = 6.630; *p* < 0.001), (d) month_6 NVS (mean difference = 5.973; *p* < 0.001), and (e) year_1 NVS (mean difference = 5.264; *p* < 0.001). Of note, post-RF NVS score started to increase after week_1 and until the end of the 12-month period, yet the differences were not significant within the first three months (week_1, month_1, and month_3), and only comparisons of these three post-RF NVS scores with month_6 and year_1 were significant (*p* < 0.05, Bonferroni correction).

**Figure 3:**
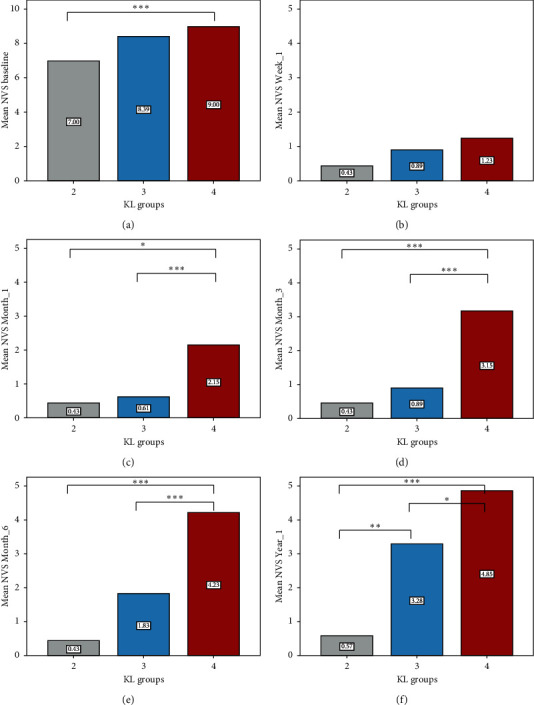
Bar chart illustrating mean NVS scores prior and during the follow-up period across the three osteoarthritis groups (KL-2, KL-3, and KL-4) (note: ^*∗∗∗*^*p* < 0.005, ^*∗∗*^*p* < 0.01, and ^*∗*^*p* < 0.05 after Bonferroni correction).

**Figure 4:**
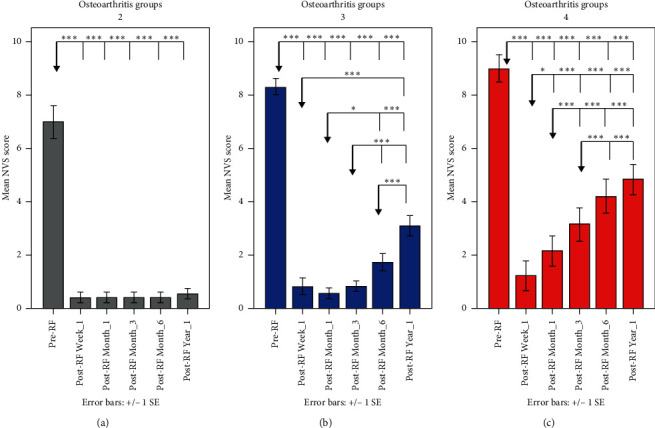
Bar charts illustrating mean NVS scores and standard error (1SE) prior and during the follow-up period within each osteoarthritis group (note: the reference point for each comparison is indicated with a grey arrow; ^*∗∗∗*^*p* < 0.005, ^*∗∗*^*p* < 0.01, and ^*∗*^*p* < 0.05 after Bonferroni correction).

Based on the significant group by time interaction, pairwise comparisons were further examined (Figures 3 and 4). We found significant differences on NVS score between KL-2 and KL-4 at baseline (*p*=0.034), month_1 (*p*=0.030), month_3 (*p*=0.001), month_6 (*p* < 0.001), and year_1 (*p* < 0.001). Osteoarthritis groups KL-3 and KL-4 differed on NVS score in month_1 (*p*=0.008), month_3 (*p* < 0.001), month_6 (*p*=0.001), and year_1 (*p*=0.023). Furthermore, groups KL-2 and KL-3 differed on NVS score only in year_1 (*p*=0.006). There were no significant differences between KL-2, KL-3, and KL-4 in week_1 (*p* > 0.05).  Figure 4 depicts the profile of NVS scores across time separately for each osteoarthritis group (KL-2, KL-3, and KL-4) as well as between different timepoint comparisons within each group. Improvement (pain decrease of more than 4 NVS units during follow-up) was noticed in 35/39 knees (89.7%) at first week, 36/39 knees (92.3%) at first month, 35/39 knees (89.7%) at three months, 32/39 knees (82.1%) at six months, and 25/39 knees (64.1%) at one year. There was no recurrence (pain increase) during the follow-up. The percentage of cases with no changes or improvement in follow-up compared to baseline is shown in Figure 4. There was not any significant association between improvement/no change and osteoarthritis group (chi-square, *p* > 0.05).

Mean pain score prior to RF was 8.31 ± 1.70 NVS units. This baseline score was reduced to a mean value of 0.90 ± 1.50 NVS units one-week post-RF, 1.08 ± 1.53 at one month, 1.54 ± 1.88 at three months, 2.33 ± 2.17 at six months, and 3.23 ± 2.23 at 12 months of follow-up (Table 2 and Figure 2). A repeated measures ANOVA with Greenhouse–Geisser correction showed that mean NVS differed significantly between timepoints *F* (2.979, 113.189) = 190.026; *p* < 0.001, partial eta2 = 0.833). Paired samples *t*-tests with Bonferroni correction were used to examine post hoc comparisons between baseline NVS and NVS at different follow-up timepoints. We found significant differences between baseline and (a) week_1 NVS (mean difference = 7.410; *p* < 0.001), (b) month_1 NVS (mean difference = 7.231; *p* < 0.001), (c) month_3 NVS (mean difference = 6.769; *p* < 0.001), (d) month_6 NVS (mean difference = 5.974; *p* < 0.001), and (e) year_1 NVS (mean difference = 5.077; *p* < 0.001).

**Figure 5:**
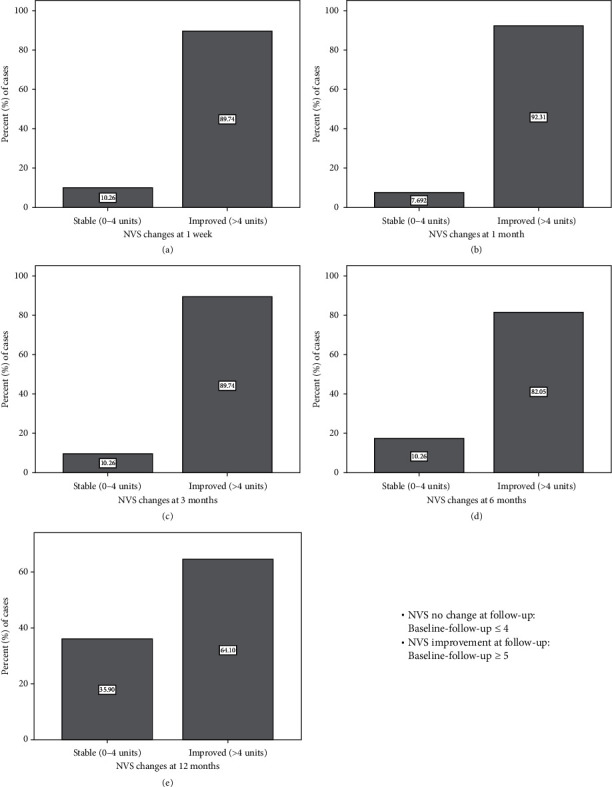
Percentage of cases with no change or improvement of pain based on the comparison between baseline (before RF) and follow-up (post-RF) NVS scores.

Improvement (pain decrease of more than 4 NVS units during follow-up) was noticed in 35/39 knees (89.7%) at first week, 36/39 knees (92.3%) at first month, 35/39 knees (89.7%) at three months, 32/39 knees (82.1%) at six months, and 25/39 knees (64.1%) at one year. There was no recurrence (pain increase) during the follow-up. The percentage of cases with no changes or improvement in follow-up compared to baseline is shown in Figure 5.

## 4. Discussion

The present study adds to the growing number of case series showing that intra-articular application of PRF is an efficacious and safe technique in terms of achieving pain reduction [7–9, 15]. Similar to other studies, in the present case series, the treatment of pain due to knee osteoarthritis with intra-articular application of PRF was successful and well tolerated [7–9, 15]. One major difference of the present study is that all patients were treated with STP PRF as a stand-alone therapy, resulting however in no significant differences concerning the efficacy and safety rates [7–9, 15]. Another major difference is that although towards the end of the 12-month period, there is a tendency for pain increase and this is significantly lower than the baseline requiring no new therapeutic session for further symptom improvement [7–9, 15].

Although the pathophysiology and action mechanism of intra-articular PRF is not entirely clear, potential explanations include modulation of inflammatory response especially associated with cytokine production along with the effect upon peripheral osseous nerve endings which are related to pain perception; this effect upon nerve fibers seems to be amplified whenever a low-energy electric field is applied within a closed joint [18–20]. There is no doubt that peri- and postprocedural pain is limited during PRF in comparison to continuous RF neurolysis, thus enabling the procedure to be held under local anesthesia. When compared to continuous RF neurolysis of genicular nerves, intra-articular application of STP PRF is a less complex procedure with shorter intraprocedural duration since only one electrode is necessary to be placed inside the joint instead of three placed at the level upper and lower medial and upper lateral genicular nerves.

Kellgrene and Lawrence scale classifies osteoarthritis based upon the severity of radiographic findings [21]. The results of the present study are in accordance with those of other paper reporting that higher grades in KL scale (more severe osteoarthritis) are related to less and of shorter duration pain reduction [7, 9]. Although in the 1^st^ week and 1^st^ month, there is no significant difference between KL grades 2, 3, and 4 later in the follow-up period at 6^th^ and 12^th^ month of follow-up and there is a clear difference between patients with severe osteoarthritis (grade 4) versus those with moderate (grade 3) or mild (grade 2). Similar differences at the same follow-up timepoints are reported between KL-2 and KL-3 grades (moderate versus mild). Possibly other therapies including either neurolysis of genicular nerves or transarterial embolization may be proven more efficient in more severe osteoarthritis; however, at the moment, there are no data available to support such a hypothesis.

Limitations of our study include that this is a retrospective study lacking a control group which will consist of patients undergoing either a sham procedure or any other local therapy. Furthermore, there was no direct comparison of intra-articular application of STP PRF either to other pulsed modes or to the extra-articular neurolysis of the genicular nerves by means of continuous RF.

Percutaneous, intra-articular application of STP PRF is an effective and safe technique for chronic pain reduction in patients with knee osteoarthritis. Results seem to be reproducible and long lasting with significant patient satisfaction at 12-month follow-up. The results of the present study do not show a clear need of repeating the session at 1 year. Further evaluation of the technique against sham trial and/or other local therapies is warranted.

## Figures and Tables

**Table 1:** Demographic characteristics of the total group of cases (*N* = 39).

Variables	Statistics			
	Mean	SD	Min	Max
Age (yrs)	71.59	11.99	37	93
Gender (M/F)	9/30			
Weight (kg)	81.66	15.72	45	140
Height (cm)	164.10	6.95	150	185
BMI	30.23	4.69	15.57	44.69

Note. SD = standard deviation; Min = minimum; Max = maximum; yrs = years; M/F = male/female; kg = kilograms; cm = centimeters. Continuous variables (age, weight, and height) are presenting as mean ± SD (min-max). Gender is presenting in absolute frequency.

**Table 2:** Descriptive statistics of NVS questionnaire in the total group of 39 cases at different timepoints.

Timepoints	Statistics			
	Mean	SD	Min	Max
*Pre-RF*				
Baseline	8.31	1.70	5	10

*Post-RF*				
Week_1	0.90	1.50	0	6
Month_1	1.08	1.53	0	5
Months_3	1.54	1.88	0	7
Months_6	2.33	2.17	0	7
Year_1	3.23	2.23	0	7

Note. NVS = numeric visual scale; RF = radiofrequency; SD = standard deviation; Min = minimum; Max = maximum.

## Data Availability

The data used to support the findings of this study are available from the corresponding author upon request.
